# Performance of a non-contact veterinary infrared thermometer and reference intervals of equine temperature at different body sites

**DOI:** 10.3389/fvets.2025.1583839

**Published:** 2025-04-17

**Authors:** Daniela Alberghina, Carlotta Tombolani, Fausto Quintavalla

**Affiliations:** ^1^Department of Veterinary Science, University of Messina, Messina, Italy; ^2^Independent Veterinary Practitioner, Verona, Italy; ^3^Department of Veterinary Science, University of Parma, Parma, Italy

**Keywords:** horses, rectal temperature, ocular temperature, gingival temperature, perineal temperature

## Abstract

Evaluating the body temperature of horses (*Equus caballus*) is essential for monitoring their health. Rectal thermometry is the most common method for determining the temperatures of adult horses and foals. However, this method requires restraining the animals for several seconds, and it poses potential risks to both horses and humans. This study compared infrared and rectal thermometry in 126 horses, evaluating age and measurement site influences on body temperature. Horses were divided into three groups: foals (4–12 months), Shetland ponies (8–12 months), and adult horses (2–30 years). Rectal, ocular, gingival, and perineal temperatures were recorded to assess the potential of non-invasive infrared methods. Reference intervals of temperature at different body sites are provided. No significant differences were found in gingival and perineal temperatures among groups. Foals showed significantly higher rectal temperatures than adults (*p* < 0.001), likely due to age. Shetland ponies showed higher ocular temperatures than foals and adult horses (*p* < 0.05, *p* < 0.001), probably because they were influenced by ambient temperature, which significantly correlated with ocular readings. Significant positive correlations existed between ocular and rectal (*p* < 0.01) and perineal and rectal temperatures (*p* < 0.0001). Bias was −0.2°C (ocular vs. rectal) and 2°C (perineal vs. rectal). Perineal temperature, despite numerical differences, correlated well with rectal temperature, allowing indirect estimation with a correction factor, and was unaffected by ambient temperature. This suggests infrared perineal temperature may be a viable rectal thermometry alternative for estimating equine body temperature, enhancing animal welfare through non-invasive methods.

## Introduction

Evaluating body temperature is a valuable tool for monitoring an animal’s physiological status and stress responses. It is also a valid method for assessing animal welfare and for the early identification of changes in clinical condition associated with various conditions in horses (*Equus caballus*), including infections, immune-mediated diseases, endocrine disorders, systemic inflammatory response syndrome, colic, dehydration, neoplasia, heat stress, shock, and increased immune response to vaccination (1–5). A horse’s body temperature is typically maintained within a narrow range, around 37.1 ± 0.5°C at rest (1). In healthy horses, daily temperature can fluctuate by an average of 0.7°C, with the lowest point usually around 9 am and the highest around 9 pm (6). This daily rhythm persists even in constant darkness (6). It’s important to note that these values can vary depending on the type of thermometer used, environmental conditions, and exercise levels (7, 8). Among the various methods for measuring body temperature, rectal temperature measurement with a digital thermometer is an established standard in horses (1). Rectal digital temperature is accurate and correlates well with core body temperature (9). However, this method requires restraining the animal, which can pose a risk to both the handler and the horse. While some horses tolerate rectal temperature measurement well, others may resist, sometimes with sudden movements. This resistance not only increases the risk of injury to both handler and horse but can also lead to inaccurate temperature readings due to stress-induced physiological changes. Finally, some studies suggest that rectal temperature may not be the most sensitive indicator of early-stage fever (10) or for post-exercise body temperature monitoring (5), and it also carries the potential risk of disease transmission and other hygiene concerns (11). Furthermore, accurate rectal temperature measurement depends on the consistent insertion depth of the thermometer (1). Less invasive body temperature evaluation in horses can lead to a less stressful experience during clinical examinations and routine health checks, ultimately improving their overall care. Non-contact infrared thermometers are widely used in human pediatric medicine, particularly with uncooperative patients (12, 13). However, the agreement between non-contact infrared thermometers and rectal thermometers in horses has only recently been investigated, with conflicting results (4, 14–16). Horses and ponies exhibit differences in their thermoregulatory responses to heat exposure, including the onset of sweating (17), suggesting that smaller equids may tolerate a wider range of body temperatures. The capacity of smaller equids to potentially tolerate a broader spectrum of body temperatures is attributable to a confluence of factors, notably their size and the consequent surface area-to-volume ratio. The detection distance and ambient temperature are critical factors for traditional infrared thermometers. However, some recent infrared thermometers incorporate automatic adjustments for measurement distance and ambient temperature, optimizing measurement reliability. A less invasive evaluation of body temperature results in a less time-consuming and easier procedure for clinicians. Infrared procedures can also be less stressful for animals undergoing clinical assessment and health monitoring, thereby improving the quality of care they receive. However, it is important to assess the correlation with rectal temperature and the influence of ambient temperature to determine if a site for infrared measurement is a suitable alternative to rectal temperature. Measuring eye temperature offers several advantages: it is a rapid, relatively easy, and accurate method, as this area is free from the interference of hair, unlike other body sites (11, 18, 19). Eye temperature, typically measured using infrared thermometry, has been evaluated in response to various stimuli, such as physiological reactions of fear and transport stress (19, 20). However, to the authors’ knowledge, its use in equines has not been validated, and reference ranges for eye temperature have not been previously established. In scientific studies and healthcare practice, it is important to have reference intervals for a given parameter. Reference intervals in veterinary medicine are commonly established based on a relatively small population of clinically healthy individuals. Reference intervals are typically calculated using a nonparametric percentile method, determining the 90% confidence interval (2.5th to 97.5th percentile) from data of at least 120 clinically healthy animals (21). This study aimed to: (1) determine the correlation between digital rectal temperature and infrared temperatures at ocular, gingival, and perineal sites in healthy horses, evaluating the influence of ambient temperature; and (2) establish reference temperature intervals for each described body site, facilitating the implementation of infrared temperature detection in research and clinical practice.

## Methods

### Ethics statement

This study was approved by the University of Parma’s Animal Ethics Committee (04/CESA/2025). All procedures were carried out following relevant Italian guidelines and regulations.

### Animal and data collection

126 horses of various breeds were enrolled in this study from three different yards in the province of Verona (Italy). Group I (*n* = 25 foals, 13 females, 12 males, aged 2–12 months), Group II (*n* = 25 Shetland ponies, 9 females, 16 males, aged 8–12 months), and Group III (*n* = 76 adult horses, 55 mares, 2 stallions, 19 geldings, aged 2–30 years; mean age 10.16 ± 6.61 years). All horses were clinically healthy at the time of measurement. Measurements were taken daily between 10:00 AM and 4:00 PM CET, September 27, 2024 – February 10, 2025.

**Figure 1:**
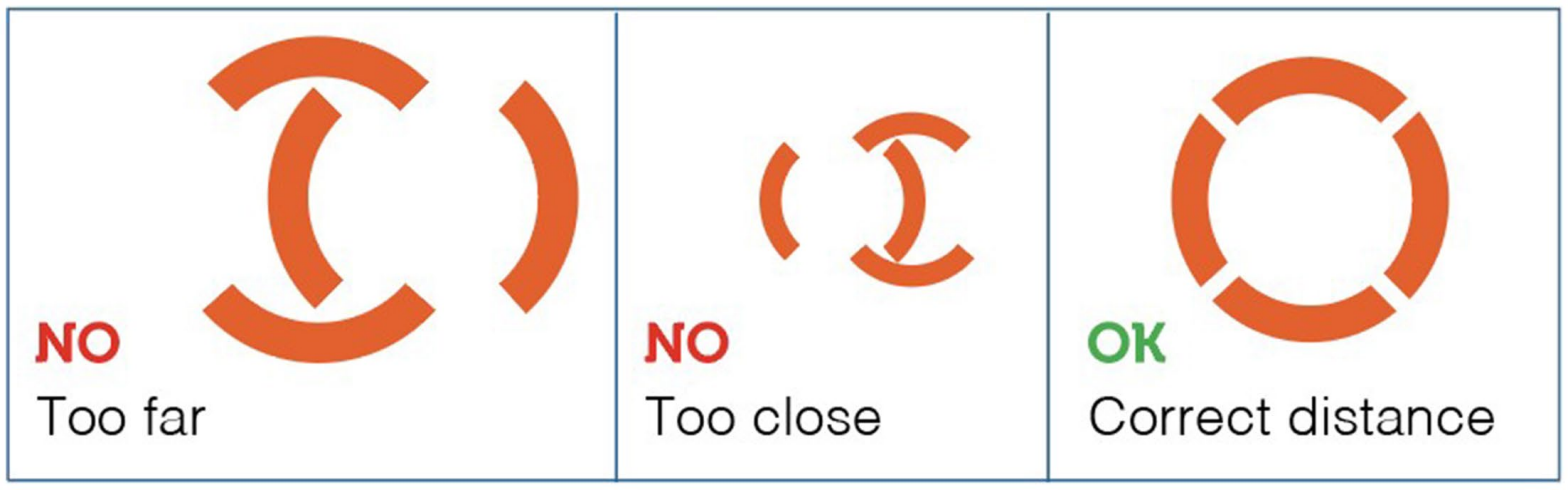
The device’s optic positioning system uses four arches in the display, which form a circle at the correct target distance.

**Figure 2:**
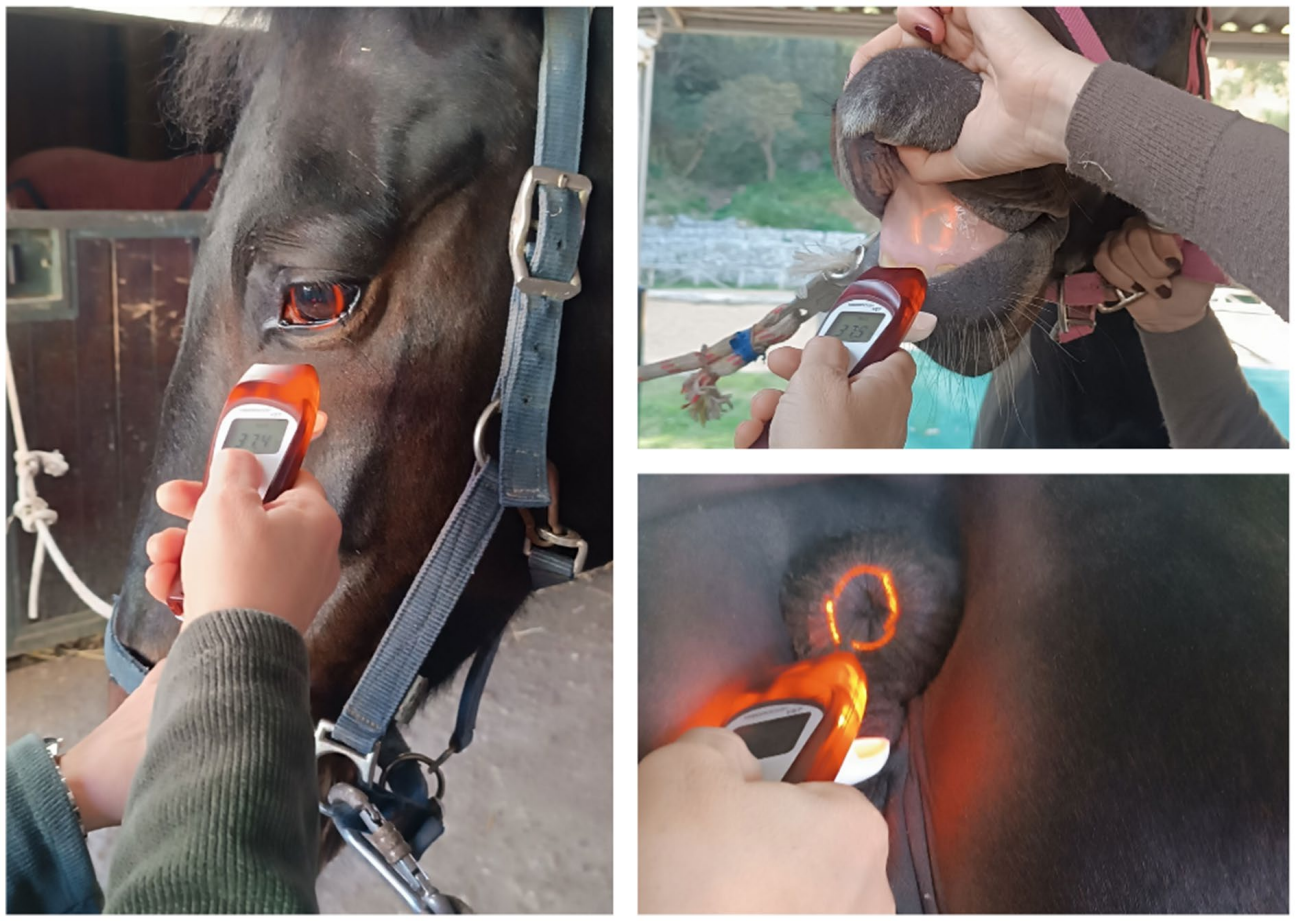
Equine temperature measurement using a non-contact veterinary infrared thermometer at three different measurement body sites.

Horses were in their normal housing environments (pens or boxes) during data collection. Familiar technicians restrained the horses with a headcollar and lead rope before taking temperature measurements. All temperature recordings were in Celsius, recorded to one decimal place. Rectal temperatures were then measured twice for each animal using a digital thermometer for large animals (Scala SC 1080, Scala Electronic GmbH Ruhlsdorfer Str. 95, Stahnsdorf, Germany, 14532). As described by Hall et al. (1), the rectal digital thermometer was inserted approximately 5 cm into the rectum, to the level of the thermometer display window, held against the rectal wall, and removed after the audible alarm indicated a stable peak temperature. The thermometer was cleaned and disinfected with alcohol before each measurement. Infrared temperature readings were taken immediately after rectal measurements. For non-contact infrared readings, a single, commercially-available device was used (Visiofocus Vet, Tecnimed srl, P.le Cocchi, Vedano O. (VA), Italy 21040). The infrared thermometer was stabilized by allowing it to equilibrate with the ambient temperature, which was measured by the device itself on a wall or ground surface shielded from direct sunlight. This stabilization process is performed using the device’s Manual Quick Calibration System. The device’s optic positioning system uses four arches in the display, which form a circle at the correct target distance (Figure 1). As illustrated in Figure 2, infrared temperature readings were taken at a distance of approximately 6 cm (automatically indicated by the device) from the central cornea region of the eye, the premaxillary gingival sulcus, and the anal triangle of the perineum. At each site, temperature readings were performed twice, with a 30-s interval between each reading, and the entire process was completed for each horse within 5 min. Data collected for each horse included the date and time, ambient temperature, rectal temperature, and infrared temperatures (recorded in the following order: perineum, eye, premaxillary gingiva).

### Data analysis

Statistical analyses were performed using GraphPad Prism v8.4.2 (GraphPad Software, Inc., La Jolla, CA, USA). Normality was assessed using the Shapiro–Wilk test and visual analysis of the data. The Shapiro–Wilk test indicated that all data were normally distributed except for ambient and ocular temperatures. Normally distributed data are presented as mean ± SD, while non-normally distributed data are presented as median. The average of the two readings for each site was used for further analysis. The effect of horse sex on temperatures was investigated using a Mann–Whitney *U* test. Group temperature comparisons were performed using one-way ANOVA with Tukey’s test for parametric data, and Kruskal-Wallis with Dunnett’s test for non-parametric data. Data were pooled to calculate reference intervals. For normally distributed data (*n* > 120), reference intervals were calculated as the mean ± 2 standard deviations. For skewed data, percentiles were used. Agreement between rectal and infrared temperatures was evaluated using Bland–Altman analysis. The relationship between ambient and body temperatures was analyzed using a gamma generalized linear model (GLM) in R, version 3.5.0 (R Foundation for Statistical Computing).^1^ The level of statistical significance was set at *p* < 0.05.

## Results

A total of 126 mean temperature measurements were collected for each site (infrared eye, infrared gingival, infrared perineal, and rectal), and these data were used to determine the reference temperature intervals for normal equine temperature at each site (Table 1). The mean ambient temperature during data collection was 15.04 ± 7.11°C. Ambient temperature during body temperature measurement was significantly higher in Group II than in Groups I and III (Dunnett’s, *p* < 0.0001). Ocular temperatures were significantly higher in Group II compared to Groups I and III (Dunnett’s, *p* < 0.05 and *p* < 0.001, respectively). Rectal temperatures were significantly higher in Group I compared to Group II (Tukey’s, *p* < 0.01) and Group III (Tukey’s, *p* < 0.001). No significant differences were found between sexes, nor among the groups for infrared temperatures measured at the gingival mucosa and perineum (Table 1). Bland–Altman analysis, using the average of the difference in measured temperature, was performed to evaluate bias and agreement between rectal and infrared temperature measurements. The eye (−0.21°C [95% CI, −1.18–0.75°C]) had the smallest bias relative to rectal temperature, followed by gingiva (1.75°C [95% CI, 0.44–3.05°C]) and perineum (2.02°C [95% CI, 0.92–2.12°C]). The relationship between ambient and body temperatures was examined using gamma regression analyses. Significant correlations, as detailed in Table 2, were identified between rectal temperature and both eye and perineal infrared temperatures, and between ambient temperature and both eye and gingival infrared temperatures. Conversely, perineal infrared temperature exhibited no correlation with ambient temperature. Notably, significant positive correlations were observed between perineal infrared and rectal temperatures (*p* < 0.001) and between eye infrared and rectal temperatures (*p* < 0.01).

**Table 1:** This table presents reference intervals for rectal and infrared temperatures in horses, along with the mean ambient temperatures (°C) measured across different groups.

Body site	Reference intervals (126)	Group I (25)	Group II (25)	Group III (76)	Intergroup comparison
Rectal (°C)	37.72 ± 0.34	37.99 ± 0.34	37.66 ± 0.20^a^	37.64 ± 0.34^b^	*F*_(2,123)_ = 10.87 *p* < 0.001^1^
Ocular (°C)	37.50 ± 0.42 (37.50–37.60)	37.56 ± 0.34	37.78 ± 0.29	37.39 ± 0.44^c^	*p* < 0.001^2^
Gingival (°C)	39.47 ± 0.62	39.44 ± 0.44	39.41 ± 0.42	39.49 ± 0.44	NS^1^
Perineal (°C)	39.75 ± 0.62	39.94 ± 0.58	39.73 ± 0.55	39.69 ± 0.65	NS^1^
Ambient	Mean values				
Temperature (°C)	15.07 ± 7.11	10.78 ± 1.27^d^	23.32 ± 3.94	13.80 ± 6.85^d^	*p* < 0.0001^2^

NS, not signicant. ^1^ANOVA and Tukey’s multiple comparison test differences vs Group I ^a^*p* < 0.01, ^b^*p* < 0.0001; ^2^ Kruskal Wallis and Dunnet’s multiple comparisons test differences vs Group II ^c^*p* < 0.01; ^d^*p* < 0.0001.

For normally distributed data (rectal, gingival, and perineal temperatures), reference intervals were calculated as the mean ± 2 standard deviations, and differences between groups were analyzed using ANOVA. For ocular temperature data, 90% confidence intervals were provided, and differences between groups were analyzed using the Kruskal-Wallis test.

**Table 2:** Gamma regression analyses examined the relationship with ambient and body temperatures.

	Explanatory variables	
Response variables	Rectal temperature	Ambient temperature
Eye temperature	*p* < 0.01	*p* < 0.001
Gingival temperature	*p* = 0.11	*p* < 0.05
Perineal temperature	*p* < 0.0001	*p* = 0.96

Specifically, infrared temperatures (response variables) were modeled using rectal and ambient temperatures as explanatory variables.

## Discussion

Infrared temperature measurements were generally well-tolerated by all horses. Gingival temperature measurement proved slightly less tolerable for the horses, probably because of the procedure required to lift the upper lip. The range intervals for rectal, ocular, gingival, and perineal temperatures in equids are provided in the present study. The mean rectal temperature observed in this study was 37.72°C with a standard deviation of 0.34°C, resulting in a temperature range of 37.38°C to 38.06°C (calculated as the mean ± 2 standard deviations). This range is broadly consistent with the previously reported normal rectal temperature range of 36.0°C-38.0°C for adult horses (1). Our average rectal temperature of 37.72°C is also very close to the 37.7°C reported by Godwin (9). The consistent insertion depth of the rectal thermometer across all three studies strengthens the comparability of these results. Since horse owners typically do not use deep rectal probes to measure their animals’ temperatures, a reference range established at a 5 cm depth is more practical for them (1). The febrile temperature in horses is generally considered higher than 38.9°C (11). However, this study did not detect temperatures exceeding 38.9°C in the rectal mucosa of the examined horses. Rectal temperature was significantly higher in foals compared to adult horses. This difference may be attributed to the foals’ increased activity during handling, or it could be related to age. Foals generally have a slightly higher normal temperature (22). In contrast to findings in humans (23) and dogs (24), age did not appear to influence ocular temperature in the horses in this study. The observed difference in ocular temperature between Groups II and III (37.78°C vs. 37.39°C, *p* < 0.01) may be attributed to the significant difference in ambient temperature between these groups (23.32°C vs. 13.71°C, *p* < 0.0001). Although there are known thermoregulatory differences between ponies and horses (17), no difference in infrared temperatures was found, at the same age, between Group I and Group II. Statistical analysis confirmed a significant correlation between ambient temperature and ocular temperature. A key limitation of this study was the disparity in ambient temperature recorded for Group II compared to Groups I and III. This study’s design, however, offers an advantage over previous research by not restricting climate conditions during infrared thermometer use. Previous studies (3, 15, 16) that employed climate-controlled conditions may not accurately reflect the variability in temperature encountered in typical clinical settings, such as barns, stables, or outdoor environments. Because as we found ambient temperature can influence ocular or gingival infrared readings, the strict climate control in previous studies may limit the generalizability of their findings to real-world scenarios where these factors are less controlled. The use of infrared thermometry in clinical practice relies on established reference intervals for normal temperatures. However, if these intervals are derived from studies conducted under specific climate conditions, they may not be applicable when ambient temperature falls outside of the controlled range. By conducting our study under more variable environmental conditions, we aimed to provide reference intervals that are more robust and applicable to the range of conditions encountered in clinical settings. Measuring eye temperature offers several advantages: it is a rapid, relatively easy, and accurate method, as this area is free from the interference of hair, unlike other body sites (11, 18, 19). Ocular temperature measurement offers the advantages of simplicity and its non-invasive nature, with ocular temperatures falling within the same range as rectal temperatures. While ocular temperature has been evaluated in response to stimuli or stress (19, 20), this study establishes the first reference intervals for equine ocular temperature. Although perineal infrared temperatures were not numerically identical to rectal temperatures, a significant correlation was observed. This study showed that subtracting 2 degrees from the perineal infrared temperature provides a reasonable estimate of rectal temperature. Perineal infrared thermometry offers a rapid and less invasive option for assessing body temperature in horses. Previous studies using the neck and forehead as infrared measurement sites reported large and inconsistent biases compared to rectal digital thermometry (15). Some authors suggest that the temperature of hairless skin may be comparable to core body temperature (25). Moreover, several previous studies were limited by inappropriate methodologies, such as using rectal thermometers not designed for large animals or performing infrared measurements through hair. Ideally, the temperature measurement should accurately reflect the core temperature in all age groups, be easy, non-invasive and harmless (26). Lastly, it should indicate the core temperature as accurately as possible without being markedly influenced by the ambient temperature (26). Rectal temperature measurement can be invasive, uncomfortable for the horse, and potentially dangerous for the handler. A previous study has monitored horse core body temperature using three methods—blood temperature (BT), rectal temperature (RT), and telemetry-based gastrointestinal temperature (GT)—finding GT to be a viable alternative (27) In our study, we did not measure core temperature, so we cannot determine which site best correlates with it. However, we observed that perineal temperature is strongly correlated with rectal temperature and is not affected by external temperature. Furthermore, it offers a non-invasive temperature detection method. Reference intervals were established for normal rectal, eye, gingival, and perineal temperatures to a wide range of ambient temperatures. Further research should investigate potential variations in normal equine temperature using non-contact infrared veterinary thermometers across diverse management settings.

## Data Availability

The raw data supporting the conclusions of this article will be made available by the authors, without undue reservation.

## Footnotes

^1^ http://www.r-project.org
